# Morphometric Study and Branching Patterns of External Carotid Artery Using Computed Tomography Angiography Among the South Indian Population: A Retrospective Study

**DOI:** 10.7759/cureus.35624

**Published:** 2023-02-28

**Authors:** Nandhini Sasikumar, Vijayalakshmi S, Gunapriya Raghunath, Balaji Karunakaran, Nithya S, Priya Dharshini KS, Kumaresan M, Sankara Narayanan G, Karthikeyan Gurusamy, Yuvaraj Maria Francis

**Affiliations:** 1 Anatomy, Panimalar Medical College Hospital and Research Institute, Chennai, IND; 2 Anatomy, Saveetha Medical College, Saveetha Institute of Medical and Technical Sciences, Chennai, IND

**Keywords:** superior thyroid artery, intra-arterial chemotherapy, endarterectomy, carotid arteries, carotid stenting

## Abstract

Introduction: The prime source of vascularization to the head and neck region is through the carotid arteries. The terminal branches of common carotid arteries, such as external carotid artery (ECA) and internal carotid artery (ICA), and their branches are crucial due to the wide area of distribution and variations in their branching pattern. The branching pattern and morphometry are essential for surgeons in the planning and execution of head and neck surgeries. Therefore, this study was conducted to observe the branching patterns of ECA and analyze them morphometrically.

Materials and methods: This retrospective study includes 100 CT images, inclusive of 32 females and 68 males. The branching pattern and luminal diameter of CCA and ECA were measured and analyzed statistically.

Results: The luminal diameter of CCA in males were as follows: 7.4 ± 1.01 (R), 7.1 ± 0.8 (L), and in females: 7.3 ± 0.9 (R), 7 ± 0.9mm (L); and the luminal diameter of ECA in males: 5.2 ± 1.0mm (R), 5.2 ± 0.9mm (L), and in females: 5.0 ± 0.9mm (R), 5.1 ± 1.0mm (L). The level of the carotid bifurcation and ECA branching pattern was observed, and variations were commonly seen in the superior thyroid artery (STA), lingual artery (LA), and facial artery (FA).

Conclusion: The findings of the present study with regard to the external carotid artery and its branching pattern correlate with previous studies. The most common variations were observed in the superior thyroid and lingual and facial arteries. Knowledge about the morphology and branching pattern of the carotid artery is essential for procedures such as intra-arterial chemotherapy, carotid artery stenting, endarterectomy, and extra-intra cranial bypass revascularization procedure where it is harvested as a donor's vessel.

## Introduction

The vascular variations in living organisms are unrestrained and widespread, mainly due to developmental changes. The carotid system distributes the blood to the head and neck region along with the vertebral system. The term carotid is derived from the Greek word ‘Kapwrides’, meaning to stupefy or throttle; ‘kapos’ also means extensive sleep. The external carotid artery (ECA), a branch of the common carotid artery (CCA), commences at the level of the upper border of thyroid cartilage [1,2]. The bifurcation level of CCA varies due to many factors such as genetic, embryological, stature, and environmental. The artery can originate at a high level or low level than normal. The head and neck receive nutrition from eight branches of ECA and the brain through its anastomosis with the branches of the internal carotid artery (ICA) and vertebral artery (VA) [3,4]. The carotid arteries are a vulnerable structure in the neck; any injury to these arteries result in complication like carotid hemorrhage [5]. The pseudoaneurysm in CCA at the level of bifurcation progress towards the ECA and its branches, disturbing the blood supply of the head and neck [6]. Frequently, diverse forms of variations are encountered in the branches of ECA, while commonly noted as in the form of thyro-lingual (TL), linguo-facial (LF), thyro-linguo-facial trunks (TLFT), auriculo-occipital trunk (AOT) and auriculo-pharyngeal trunk (APT) [7,8]. Numerous studies were carried out in ECA, and their variations were reported from various countries like Turkey, Italy, Kenya, Spain, Japan, and Brazil based on cadaveric and radiographic imaging [9-14]. The knowledge about the branching pattern and variant morphometry of ECA is very important in many radiological and surgical procedures such as intra-arterial infusion chemotherapy, chemoembolization, head and neck tumors, carotid stenting, carotid angiography, carotid endarterectomy, thyroidectomy, and revascularization procedure (as a donor vessel for extra and intra-cranial bypass surgery) [15-17]. The unknown variations in the origin of ECA and its branching pattern are one of the main causes of mortality and morbidity during the planning and execution of surgical procedures. The present study, therefore, aims to trace the anatomic variations in the morphometry, origin, and branching pattern of ECA and the vertebral level of CCA bifurcation using CT-scanned images in the South Indian population.

## Materials and methods

This retrospective study was conducted in the Department of Anatomy and Radiology and Imaging Sciences, Saveetha Medical College and Hospital, from December 2021 to July 2022. The study was initiated after obtaining the clearance of the institutional ethics committee (IEC) with the reference number SMC/IEC/2021/07/013. Inclusion criteria: Adults aged 18 years or above, patients who have undergone contrast CT angiography of the neck, patients with no history of surgeries on the external carotid artery and its branches, and patients with no history of head and neck cancer and benign tumors. Exclusion criteria: Pregnant patients, patients with significant renal impairment, patients with a history of allergic reactions to contrast dye, and patients with metallic implants or devices that may interfere with the CT scan image. The following parameters were considered for this study: bifurcation level and luminal diameter of CCA, length and luminal diameter of ECA, the origin of superior thyroid, lingual and facial arteries, and distance also calculated from the bifurcation of CCA. The data were collected and analyzed from 100 CT-scan angiography images (32 females and 68 males) by measuring the above parameters in intellispace portal 9.0 software (Phillips, Amsterdam, Netherlands). The data were statistically analyzed using the two-tailed student's t-test in Microsoft Excel 2019 (Redmond, USA) and represented by mean ± SD.

## Results

A total of 100 CT scans (68 male and 32 female) were analyzed as the material of this research.

Bifurcation level and luminal diameter of CCA: The most common level of bifurcation was observed at the level of C4 (50%), and the least common was at C2 (2%). Only 8% of cases showed a normal level of bifurcation at C3-C4. The remaining high and low levels of bifurcation are expressed in Table 1. No significant differences were observed between the genders. In addition, the luminal diameter of the CCA was measured 1 cm distal to the level of bifurcation. The mean values in males on the right and left sides were 7.4 ± 1.01 mm and 7.1 ± 0.8 mm, respectively. Similarly, in females, the mean values on the right and left sides were 7.3 ± 0.9 and 7 ± 0.9 mm, respectively. The data was evaluated based on sex and sides; the right side is higher with statistical significance compared to the left (p=0.0412).

**Table 1:** Morphometric parameters of the common and external carotid arteries and its branches

Parameter	Male		Female		P-Value
	Right	Left	Right	Left	
Common Carotid Artery	7.4± 1.01mm	7.1±0.8mm	7.3±0.9mm	7±0.9mm	0.041255932
External Carotid Artery	5.2±1.0mm	5.2±0.9mm	5.0±0.9mm	5.1±1.0mm	0.565029617
Superior Thyroid Artery	4.5 ± 1.6 mm	4.22 ± 2.8 mm	5.42 ± 3.5 mm	4.8 ± 2.1 mm	0.144982999
Lingual Artery	15.6 ± 6.6 mm	15.3 ± 5.6 mm	16.2 ± 6.5 mm	16.83 ± 4.5 mm	0.012763035
Facial Artery	22.6 ± 7.4 mm	28.7 ± 6.5 mm	23.4 ± 4.5 mm	24.7 ± 5.2 mm	0.089302
Occipital Artery	19.1 ± 10.6 mm	19.4 ± 10.5 mm	19.3 ± 9.5mm	18.3 ± 7.3 mm	0.410149168

1. Length and Luminal diameter of ECA: The luminal diameter of the ECA was measured 1 cm distal to the common carotid bifurcation, and the mean values in males on the right and left sides were 5.2 ± 1.0 mm and 5.2 ± 0.9 mm, respectively, whereas the mean values in females on the right and left sides were 5.0 ± 0.9 mm and 5.1 ± 1.0 mm. The p-values were statistically significant between the genders on both sides (p=0.5650). The length of the ECA was taken from the bifurcation of the CCA to the neck of the mandible. The mean value in females on the right and left sides were 78.4 ± 0.6 mm and 77.2 ± 0.3 mm, respectively. Whereas in males, 80.3 ± 0.4 mm and 79.3 ± 0.9 mm on the right and left sides, respectively.

Origin of superior thyroid artery (STA)

The distance between carotid bifurcation to the origin of the superior thyroid artery was measured. The mean value in males on the right and left sides were 4.5 ± 1.6 mm and 4.22 ± 2.8 mm, respectively. Whereas in females, the distance was 5.42 ± 3.5 mm and 4.8 ± 2.1 mm on the right and left sides, respectively. The p-values were statistically significant between the genders on both sides (p= 0.1449). In addition, the origin of STA was also observed as 77% from ECA, 15% from CCA, and the remaining 8% from CCB.

Origin of lingual artery: The distance between Carotid artery bifurcation and the origin of the lingual artery was measured. The mean values in males on the right and left side were 15.6 ± 6.6 mm and 15.3 ± 5.6 mm, respectively. Whereas in females, the mean values on the right and left side were 16.2 ± 6.5 mm and 16.83 ± 4.5 mm, respectively. The p-value (p=0.0127) shows that the right side is more statistically significant than the left. In addition, this study also observed the origin of LA, i.e., 80% from ECA, 1% from the TLF trunk, 4% from the TL trunk, and 15 % from the LF trunk.

Origin of facial artery: The vertical distance between carotid artery bifurcation to the origin of the facial artery was measured, and the mean values in males were 22.6 ± 7.4 mm and 28.7 ± 6.5 mm on the right and left side, respectively. Whereas in females, the mean values were 23.4 ± 4.5 mm and 24.7 ± 5.2 mm on the right and left sides, respectively. The p-value (p=0.0893) is statistically significant between the sexes on both sides. 84% of FA took origin from ECA and 15% from LF trunk, and 1% from TLF trunk.

Origin of occipital artery: The vertical distance between the carotid artery bifurcation to the origin of the occipital artery was measured, and the mean values in males were 19.1 ± 10.6 mm and 19.4 ± 10.5 mm on the right and left side, respectively. Whereas in females, the mean values were 19.3 ± 9.5 mm and 18.3 ± 7.3 mm on the right and left sides, respectively. The p-value (p=0.4101) is statistically significant between the sexes on both sides. No variation was observed in the occipital artery.

**Figure 1:**
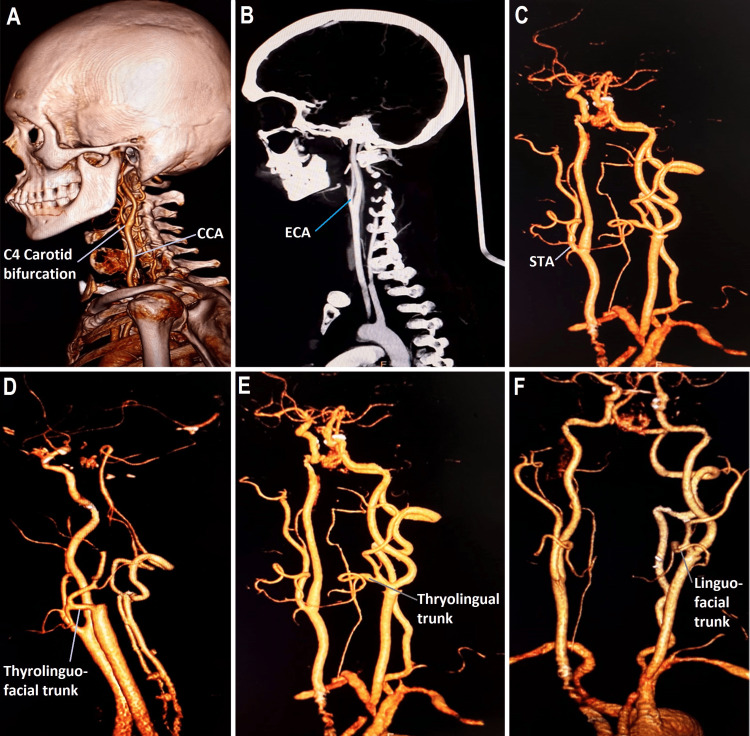
Shows the variations observed in the origin of the common carotid artery and the branching pattern of the external carotid artery A. High level carotid bifurcation, B. Normal level carotid bifurcation, C. Superior thyroid artery from carotid bifurcation, D. Thyrolinguofacial trunk, E. Thyrolingual trunk, F. Linguofacial trunk

The variations observed in the present study and morphometric parameters are shown in Figure 1 and Table 1.

## Discussion

The purpose of this research was to evaluate and discuss the levels of CCA bifurcation and the morphometry of common carotid arteries. And also to analyze the variations in the origin of branches of ECA using CT images compared with the previous literature. The findings of this study revealed that the CCA bifurcation was noted in different vertebral levels, and the variant origin of different branches of ECA was observed, especially the origin of STA, FA, and LA. The variations in humans are unrestrained in nature due to numerous reasons like genetic, nutritional, developmental, and environmental factors [18]. The developmental variations are quite common in blood vessels, nerves, bones, organs, and almost all tissues in the body. Even though wide variations occur in humans, it alters the anatomical and physiological roles of the body [19]. The variations in the arteries and veins are common, and it could be due to abnormal angiogenesis as a result of oxygen demand which leads to the stabilization of hypoxia-inducible factor 1-A, which upregulates vascular endothelial growth factor (VEGF) and NOS expression [20]. The production of nitric oxide synthase dilates the existing blood vessels and extravasation of plasma proteins, leading to protease and matrix metalloproteinase expression. The plasma proteins play a major role in the proliferation and angiogenesis of endothelial cells, which leads to the formation of abnormal vasculature [20]. The variations in vasculature enhance the interest among anthropologists, anatomists, radiologists, and surgeons. Sound knowledge about the normal branching pattern and bifurcation level of ECA with its variations is crucial while handling patients in both medical and surgical aspects like angiography, thyroidectomy, laryngectomy, and head and neck tumors.

The knowledge regarding the bifurcation level of CCA is crucial in surgeries of head and neck areas to avoid vascular damage during catheterization of carotid arteries and intra-arterial administration of chemotherapeutic agents [21,22]. Hence the present study was carried out to analyze the bifurcation levels and luminal diameter of CCA among the genders using CT images and to correlate with the previous studies. Normally, the CCA terminates at the upper border of thyroid cartilage or between the C3-C4 vertebra into ECA and ICA [1]. The findings of the present study revealed that only 8% were at the normal level, remaining bifurcated at various vertebral levels. The most common level of bifurcation is seen at the level of C4 (50%), followed by C3 (34%), C4-C5 (6%), and C2 (2%). The results of this study correlated with Padma Badhe and Furukawa s et al. done in CT [23,24]. In contrary previous studies showed that there is no bifurcation of CCA at the normal levels, and the most common level was observed at the C4 level, followed by C3 levels [25]. Similarly, many cadaveric studies were done, and their normal level of bifurcation was observed by various authors: 75% [26], 63.15 [27], 48.3% [28], 56.76% [29], 57.5% [30], 22.5% [31], 39% [32], 57% [33], 32.14% [34], 77% [35], 68.72% [36], and 83.75% [37]. In addition, the high and low level of bifurcation was also noted. In the present study, high-level and low-level bifurcation is noted in 36% and 56% of CT images, respectively. Similarly, many studies were done in cadaver and CT images, 24.75% [26], 48.3, 5% [28], 63.8%, 13.75% [31], 42%, 1% [33], 8%, 5% [35], 37.5% [36], and 46.5%, 42.3% [38].

High-level bifurcation has more tendencies in the formation of an embolus in CCA than in ECA which can result in a stroke [8]. In addition to the level of CCA bifurcation, the luminal diameter of CCA was also observed. The mean diameter in males and females on the right and left sides were 7.4 ± 1.01 mm, 7.3 ± 0.9 mm and 7.1 ± 0.8 mm, 7 ± 0.9 mm, respectively. The right side is more statistically significant than the left side (p >0.05). The findings correlated with the previous literature [39-51]. The data of the present study compared with previous literature are shown in Table 2.

**Table 2:** Bifurcation levels of common carotid artery among various postulation

Author & Year	Population	Type of study	High level			Normal level	Low level		
			C2	C2-C3	C3	C3-C4	C4	C5	C6-C7
Anangwe D. 2008 (31)	Kenyan	Cadaver	12.5	12.5	38.8	22.5	7.5	2.5	3.75
Ambali Manoj. 2012 (33)	North Indian	Cadaver	-	-	42	57	1	-	-
Al-Rafiah A. 2011 (28)	Saudi	Cadaver	-	3.3	25	18.3	48.3	5	-
Blanca M and Eva B. 2015 (41)	Spain	Cadaver	-	-	36.85	63.15	-	-	-
Chauhan YS. 2013 (42)	North Indian	Surgical study	-	-	50	-	50	-	-
Deser SB. 2020 (43)	Turkish	CT angiography			22.7	29.5	42	7.4	
Jitpun E. 2019 (44)	Thailand	CT	0.5	11.5	19	32	21	13.5	2.5
Kurkcuoglu. 2015 (45)	Turkish	Angiography	4.9	3.5	38	32	12	8	3.1
Kishve PS. 2011 (46)	North Indian	Cadaver	-	2	-	-	-	-	-
Lo A. 2006 (32)	Newzealand	cadaver	-	55	-	39	-	5	-
Namakasa Hayashi. 2005 (47)	Japan	Cadaver	-	31.2	-	57.5	-	11.3	-
Padma Badhe. 2018 (23)	North Indian	CT Angiogram	3	4	48	28	16	-	4
Radha K. 2014 (37)	South Indian	Cadaver	-	-	11.25	83.75	-	5	-
RIBEIRO RA. 2008 (48)	Brazil	Cadaver	30		10	10	50		
Sulabha Hanumant Deshpande. 2015 (49)	North Indian	Cadaver	1	4	12	30	35	12	6
Salih Gulsen. 2009 (50)	Korean	CT image	-	-	-	-	1	-	-
Arumugam S. 2020 (51)	North Indian	Cadaver	16	24	60	-	-	-	-
Usha Chalise. 2021 (36)	Nepal	Cadaver	-	5.5	25	63	8.3	-	-
Woldeyes DH. (38)	Ethiopia	Cadaver	-	46.15	-	53.85	-	-	-
Present study 2023	Indian	CT Angiography	2	8	-	-	50	-	-

Normally, the ECA terminates at the level of the neck of the mandible into superficial temporal and maxillary arteries. The average length of the ECA in males was 80.3 ± 0.4 cm, 78.4 ± 0.6 cm, and in females, 79.3 ± 0.9 cm, 77.2 ± 0.3 cm on the right and left sides, respectively. The results of this study in the south Indian population were consistent with the north Indian population [52], the Nepalese population [53], and the Japanese population [13]. In contrast, these two studies were done in cadavers, while the present study was done using CT images. Apart from the length, the luminal diameter of ECA was evaluated. The findings of the present study showed that the luminal diameter was higher on the right side than the left side, but it was not statistically significant, while in comparison with the gender, the males had larger diameters than the females and showed statistically significant differences.

The variations in the origin of ECA branches are frequent, and it could be due to various factors. In the present study, the variations were noted only in the following arteries, such as STA, facial artery, and LA, remaining arteries showed a normal pattern.

The superior thyroid artery (STA) is the first branch of the external carotid artery and plays a vital role in carrying nutrition to the thyroid gland [54]. The detailed knowledge of variations in the STA are essential for ENT surgeons while performing various surgeries in the head and neck, such as tumor removal, emergency cricothyroidotomy, embolization, radical neck dissection, catheterization, aneurysm reconstruction, and carotid endarterectomy. In addition, this artery is used as a recipient artery in microvascular free tissue grafting [55,56]. The standard medical textbooks expressed that STA takes origin from the ventral aspect of ECA, while various CT and cadaveric studies reported huge variations in the origin of STA [57]. Frequently, STA originates from any one of the following sites of carotid systems such as from the stem of CCA [57-61], at the bifurcation of CCA [57,59], from the stem of ICA [62,63], from the subclavian artery [58] and from the LF trunk [58]. The findings of this study showed that the origin of STA was 77% (ECA), 15% (CCA) and 8% (CCAB) observed from CT images and were expressed in Table 3. Our results were similar to previous studies in Mexican [64], Turkish [65], India [66] (Table 3). The detailed understanding of the vascularity of STA to the thyroid gland is crucial to surgeons to prevent any alarming hemorrhage while treating thyroid disease.

**Table 3:** Various sites of origin of superior thyroid artery STA: Superior Thyroid Artery, CCA: Common Carotid Artery, CB: Carotid Bifurcation, ECA: External Carotid Artery.

Authors and years	Population studied	Sample size (n)	Type of study	Common site for origin of STA		
				CCA	CB	ECA
Anjalee Govindrao ovhal et al., 2016. [67]	Indian	60	Cadaver	3.33% (Right) 8.33% (Left)	-	76%
Aby.s Charles et al., 2020. [52]	Indian	30	Cadaver	43.33%	13.33%	43.33%
Esen K 2017 [65]	Turkish	640	CT angiography	14.1%	20.5%	64.5%
Herrera nunez M et al., 2020 [64]	Mexican	152	CT angiography	37.5%	-	50.7%
Manjunath C S, 2016 [68]	Indian	30	Cadaver	16.66%	23.34%	60%
Pankaj Gupta 2014 [66]	Indian	25	CT angiography	7%	21.5%	71.5%
Veena Vidya Shankar et al., 2017 [69]	Indian	80	Cadaver	31.25	15%	53.75%
Present study	Indian	100	CT angiography	15%	8%	77%

In 80% of cases, the lingual artery (LA) arose from the ECA, and the remaining originated in diverse forms, such as the LF trunk (15%), the TL trunk (4%), and the TLF trunk (1%). Similarly, many cadaveric studies expressed the diverse variation in the LA origin. In the Turkish population observed that the LA arises from the LF trunk (20%), TL trunk (2.5%), and TLF trunk (2.5%) [70]. In the Kenyan population, the origin of LA was observed from the LF trunk (44.7%) [71]. In the Brazilian population LFT, 19.4% and TFT 2.5 % [72], and Kenyan population LFT, 24. 29%, TFT, 5.71% [73], In the Bengali population, LFT - 6.7%, TLF -3.3 [74] (Table 4).

**Table 4:** Origin of various sources of the lingual artery TLT: Thyro-lingual trunk, TLFT: Thyro-linguo-Facial Trunk, LFT: Linguo-Facial Trunk.

Authors and years	Population studied	No. of cases	Type of Study	Common trunks		
				TLT	TLFT	LFT
Anjalee Govindrao Ovhal et al., 2016 [67]	Indian	60	Cadaver	3.33% (Right) 5% (Left)	0.83%	28.33% (Right) 30% (Left)
Deepa Devadas et al., 2017 [7]	Indian	40	Cadaver	-	1%	20%
Herrera Nunez M et al., 2020 [64]	Mexican	152	CT angiography	10.5%	-	14.5%
Ozgur Z et al., 2008 [75]	Turkish	40	Cadaver	2.5%	-	7.5%
Ogeng’o JA et al., 2015 [71]	Kenyan	224	Cadaver	-	-	44.7%
Sanjeev et al., 2010 [29]	Indian	37	Cadaver	2.7%	-	18.92%
The present study, 2023	Indian	100	CT angiography	4%	1%	14%

The knowledge regarding the variations of the lingual artery will be helpful during intra-arterial chemotherapy for the treatment of carcinoma of the tongue, flap for partial tongue reconstruction, and super selective intra-arterial chemotherapy for head and neck carcinomas [70]. In addition, trans-oral robotic surgery (TORS) should keep in mind while encountering the lingual artery ligate in various endoscopic hemoclips to prevent life-threatening bleeding in the post-operative setting. In the case of hemostasis, compressing the neck at the level of the superior thyroid cornu to slow bleeding may be performed, especially if the artery is inadvertently transected before clips are applied.

## Conclusions

The findings of the present research with regard to the external carotid artery and its branching pattern correlate with previous studies. In addition, we have compared and reviewed our data with data from various regions across the globe. The knowledge about the variations in the branching pattern of the ECA and its morphometry will be imperative to the head and neck surgeons, vascular surgeons, and radiologists while undertaking diagnostic and surgical management.
